# Ashwagandha Derived Withanone Targets TPX2-Aurora A Complex: Computational and Experimental Evidence to its Anticancer Activity

**DOI:** 10.1371/journal.pone.0030890

**Published:** 2012-01-27

**Authors:** Abhinav Grover, Rumani Singh, Ashutosh Shandilya, Didik Priyandoko, Vibhuti Agrawal, Virendra S. Bisaria, Renu Wadhwa, Sunil C. Kaul, Durai Sundar

**Affiliations:** 1 Department of Biochemical Engineering and Biotechnology, Indian Institute of Technology (IIT) Delhi, Hauz Khas, New Delhi, India; 2 National Institute of Advanced Industrial Science & Technology (AIST), Central 4, Tsukuba, Ibaraki, Japan; 3 Graduate School of Life & Environmental Sciences, University of Tsukuba, Ibaraki, Japan; 4 Supercomputing Facility for Bioinformatics and Computational Biology, Indian Institute of Technology (IIT) Delhi, Hauz Khas, New Delhi, India; University of Saarland Medical School, Germany

## Abstract

Cancer is largely marked by genetic instability. Specific inhibition of individual proteins or signalling pathways that regulate genetic stability during cell division thus hold a great potential for cancer therapy. The Aurora A kinase is a Ser/Thr kinase that plays a critical role during mitosis and cytokinesis and is found upregulated in several cancer types. It is functionally regulated by its interactions with TPX2, a candidate oncogene. Aurora A inhibitors have been proposed as anticancer drugs that work by blocking its ATP binding site. This site is common to other kinases and hence these inhibitors lack specificity for Aurora A inhibition in particular, thus advocating the need of some alternative inhibition route. Previously, we identified TPX2 as a cellular target for withanone that selectively kill cancer cells. By computational approach, we found here that withanone binds to TPX2-Aurora A complex. In experiment, withanone treatment to cancer cells indeed resulted in dissociation of TPX2-Aurora A complex and disruption of mitotic spindle apparatus proposing this as a mechanism of the anticancer activity of withanone. From docking analysis, non-formation/disruption of the active TPX2-Aurora A association complex could be discerned. Our MD simulation results suggesting the thermodynamic and structural stability of TPX2-Aurora A in complex with withanone further substantiates the binding. We report a computational rationale of the ability of naturally occurring withanone to alter the kinase signalling pathway in an ATP-independent manner and experimental evidence in which withanone cause inactivation of the TPX2-Aurora A complex. The study demonstrated that TPX2-Aurora A complex is a target of withanone, a potential natural anticancer drug.

## Introduction

The most crucial step in a cell cycle progression is the proper segregation of sister chromatids during mitosis. This process involves the formation of a highly dynamic bipolar array called mitotic spindle through the reorganization of the interphase microtubules. In animal cells, the separation of centromeres initiates this spindle assembly process that involves changes in the functional properties of many microtubule-associated proteins [1], [2]. In several cases, these changes are caused by reversible phosphorylation. Many protein kinases and phosphatases show at least transient association with either centrosomes or spindle microtubules [3]. The Aurora A kinase is a similar Ser/Thr kinase that has been found to play critical role during mitosis and cytokinesis [4], [5], [6]. It mainly associates with the centrosome and the spindle microtubules during mitosis, and functions in centrosome maturation, spindle assembly, maintenance of spindle bipolarity, and mitotic checkpoint control.

Regulation of Aurora A protein is a complex mechanism that involves an interaction between the small GTPase Ran and the spindle protein TPX2 [7], [8]. Firstly, TPX2 (targeting protein for Xenopus kinesin-like protein 2) is released by GTPase Ran from importins α and β. The binding of TPX2 to Aurora A causes the activation of the kinase by stimulating its autophosphorylation and thereby protecting it from the inhibitory action of PP1 [9], [10]. Furthermore, TPX2 binding targets the protein to spindle microtubules at the pole [11]. The human Aurora A gene, STK15 is present in the genomic region (20q13.2) and is often amplified in certain cancers [12]. Enhanced activity of Aurora A protein has been reported in several cancer types like prostate [13], ovarian [14], bladder [15], gastric [16], laryngeal [17], pancreatic [18] and breast carcinoma [19]. Constitutive overexpression of an active mutant of Aurora A caused neoplastic transformation in rat1 cells, thus indicating that Aurora A is an oncogene [20]. Furthermore, its interaction with tumour suppressors such as p53, BRCA1 and LATS2 also suggests its connection to oncogenesis. TPX2 is also a proliferation-associated protein that was found to be over-expressed in BPDE-transformed human bronchial epithelial (16HBE-C) cells [21]. It is overexpressed in liver, lung, prostate and pancreatic cancers [22] and has been identified as a candidate oncogene on 20q11.2 showing copy number-driven over-expression in non-small-cell lung cancer and PDAC [23]. TPX2 targeting siRNAs caused cell cycle arrest and apoptosis in cancer cell lines [24]. A siRNA library based screening revealed that TPX2 is one of the three genes that significantly reduced the survival of multiple human tumor cell lines [25].

In the recent past, several Aurora A kinase inhibitors have been developed as potential anti-cancer drugs. Specificity of such drugs has been a major issue that needs to be validated in experimental and clinical settings. While the mainstream approach focuses on the blocking of ATP-binding site of Aurora A kinase by the inhibitor, an alternate way would be to block its binding to the co-factors or other proteins that mediate its function independent to its ATP binding. Most of the Aurora A inhibitors identified so far block the ATP binding site of all the protein kinases and not particularly of Aurora A [26], and hence pose complexity for their clinical trials and use. Three novel Aurora kinase inhibitors have recently been described i.e., Hesperadin [27], ZM447439 [28] and VX-680 [29]. All these three drugs have been designed to target the ATP-binding site of Aurora kinase, so they inhibit all the three Aurora kinase family members- Aurora A, B and C showing a similar phenotype when tested in cell-based assays [26]. Since TPX2-Aurora A complex has been predicted to have oncogenic properties [30], an alternate approach to interfere with the ability of Aurora A to interact with TPX2 may hold promise to identify novel anticancer drugs.

We had previously identified anticancer activity in the alcoholic extract (i-Extract) of Ashwagandha (*Withania somnifera*) leaves [31], [32] and elucidated the mode of actions of its pharmacologically active metabolites [33], [34], [35], [36]. By cell-based assays linked to the chemical analysis of the plant extract, we demonstrated that the i-Extract and its component withanone kill human cancer cells selectively, mediated by activation of tumor suppressor proteins and ROS pathway [31], [37]. In order to investigate the cellular targets for anticancer activity of i-Extract and withanone, we performed loss-of-function screening using siRNA and randomized ribozyme libraries [31], [37], and identified candidate proteins. In the screening using ribozymes, we identified TPX2 as a candidate cellular target. TPX2 knockdown by siRNA indeed resulted in increased survival of cancer cells subsequent to the treatment with i-Extract [37]. Since TPX2 function is closely linked to Aurora A (TPX2-Aurora A complex being oncogenic), we undertook a joint computational and experimental approach to predict and validate the effect of withanone (an active anticancer component in i-Extract) on TPX2-Aurora A complex.

## Results and Discussion

**Figure 1:**
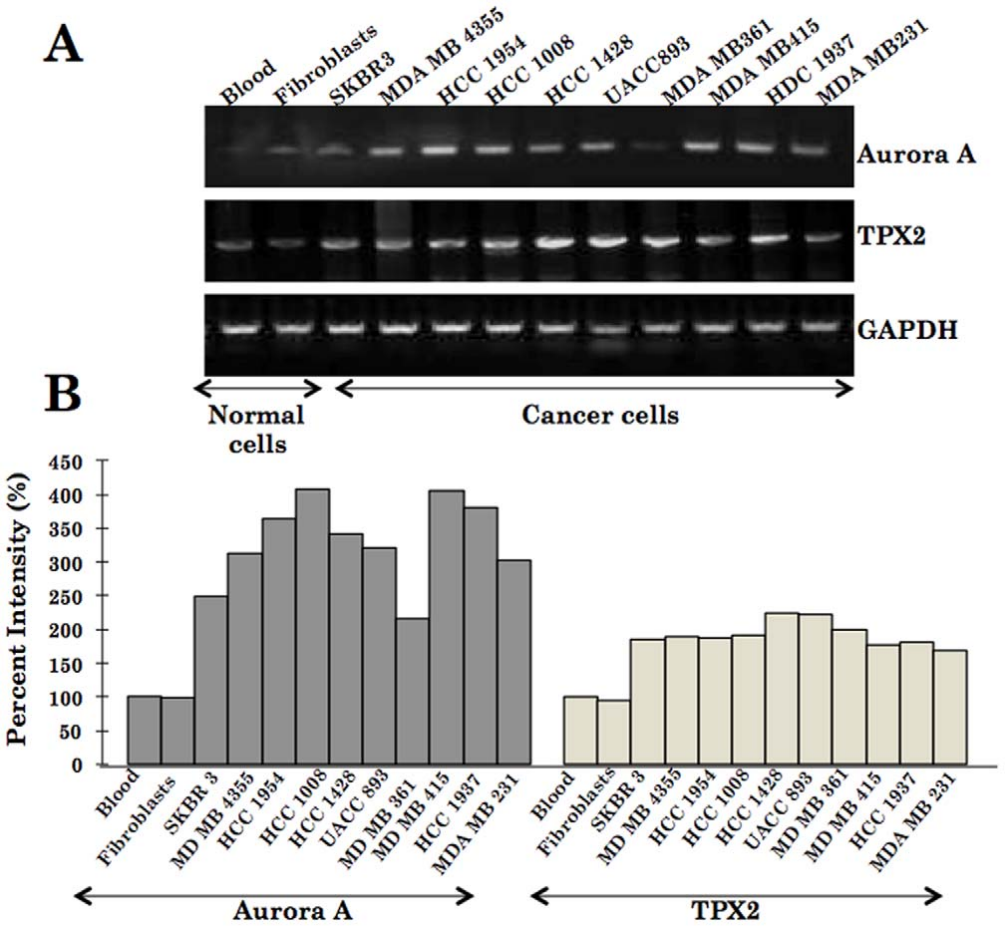
mRNA expression of Aurora A and TPX2 as analysed by RT-PCR is shown. Blood and fibroblasts represent the normal samples whereas the others are the cancer cell lines as indicated (A). Quantitation of the signals obtained by RT-PCR shown in B signified increase both in TPX2 and Aurora A in cancer cell lines.

Human gene loci, 20q11 and 20q13, that harbor TPX2 and Aurora A genes are often amplified in tumors [38], [39]. We investigated the status of Aurora A and TPX2 in 19 human cancer cell lines, 4 kinds of normal human cells along with 15 healthy subjects by PCR using gene specific primers. Both TPX2 and Aurora A were found to be amplified in large majority of cancer cells (Figure 1 and data not shown).

### Semi-flexible docking of withanone into TPX2-Aurora A

**Figure 2:**
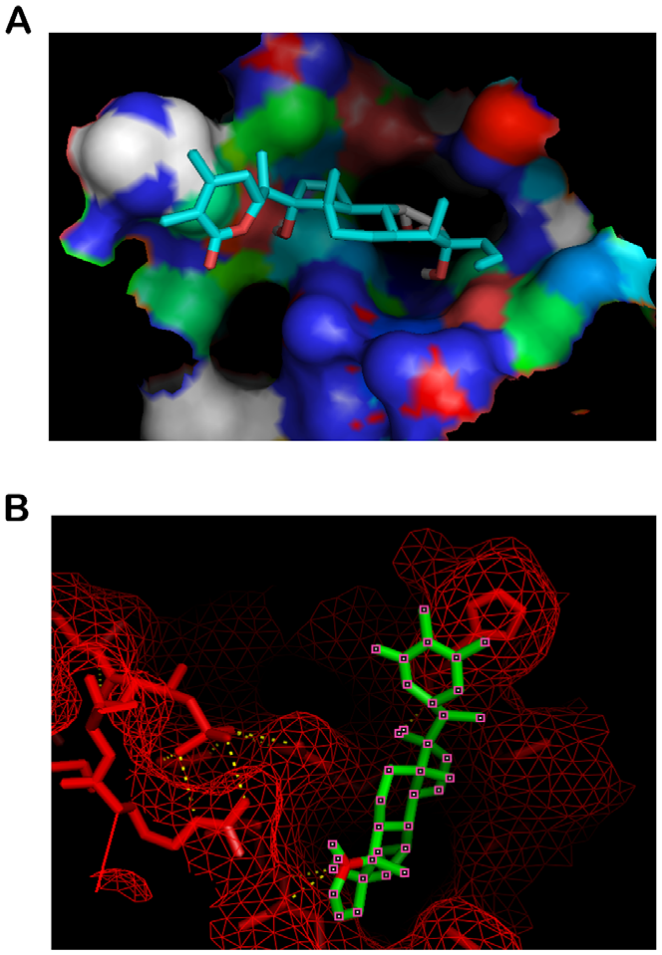
Docking representations of Withanone. (A) Ligand docked into the receptor cavity (B) Docked Ligand inside the pocket of receptor mesh.

In order to explore the possibility of disruption of the complex between Aurora A and TPX2, we first carried out semi-flexible molecular docking studies with the protein crystal structure. Before docking, the structures of receptor macromolecules were minimized in Steepest Descent followed by Conjugate Gradient methods using Accelrys Discovery studio. Figure 2A shows the docked ligand withanone inside the interfacial cavity of the two domains. Withanone gets buried inside the pocket as depicted by mesh representation in Figure 2B. For this particular configuration the binding energy of the ligand with receptor is −7.18 Kcal/mol.

**Figure 3:**
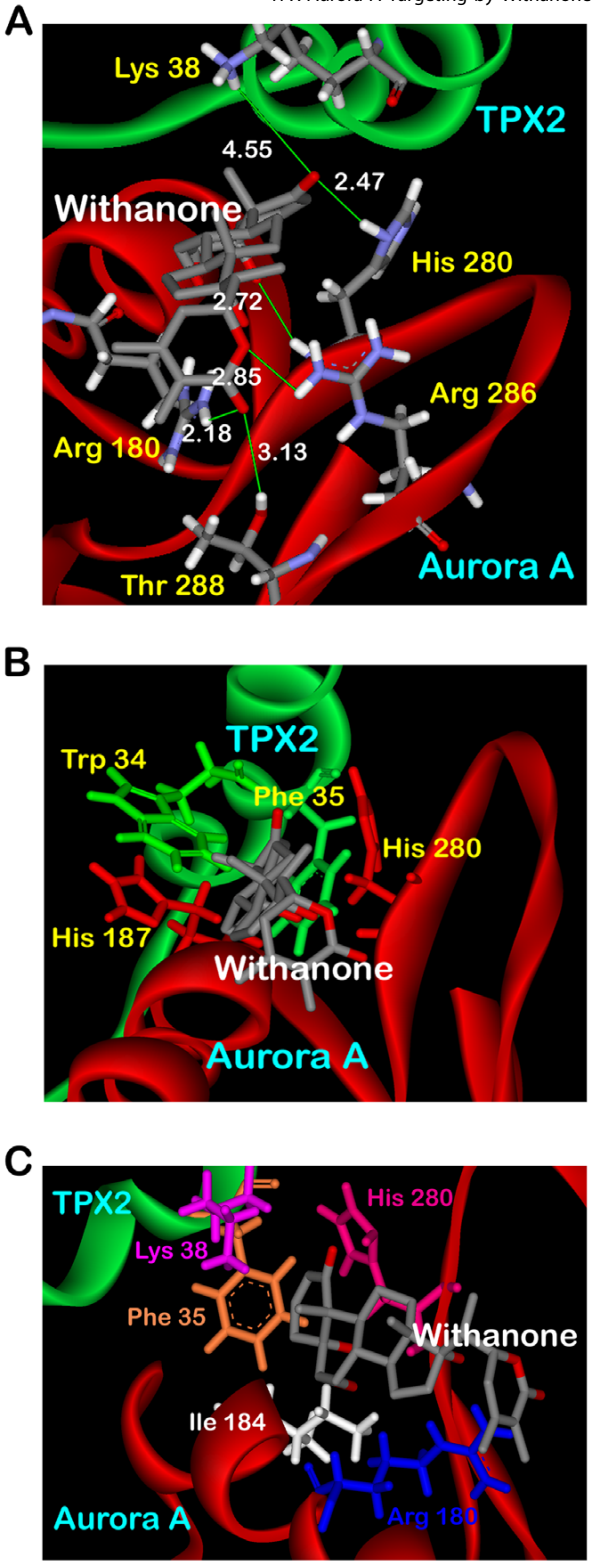
Interactions of docked withanone with rigid Aurora A/TPX2 receptor. (A) H-Bond interactions of the docked ligand with the macromolecule residues (B) Docked withanone disrupting the crucial non-covalent interactions already present in the undocked protein (C) Withanone forming hydrophobic interactions with the receptor.

The binding of withanone to the association complex is characterized by H-bonding between a terminal carboxyl group of the ligand and the side chain His 280 of Aurora A. Side chain amino hydrogens of Lys 38 of TPX2 were also found H-bonding with this carboxyl terminal of withanone (Figure 3A). The lengths of the two H-bonds formed are 2.47 and 4.55 Å. The residue His 280 of Aurora A has been reported as one of the critical residues being involved in interacting with Phe 35 of TPX2 [40]. The other end of withanone also forms H-bond with the side chain hydroxyl group of Thr 288 and amino group of Arg 180 with bond distances of 3.13 and 2.18 Å, respectively. It has also been reported that the residues Trp 34 and Phe 35 of TPX2 are responsible for intermolecular hydrophobic interactions with His 187 and His 280 of Aurora A. In the present docked structure, withanone is found disrupting the van der Waals interactions present in the association domain (Figure 3B). It was also observed that withanone sets up its own interactions with Phe 35, Lys 38 of TPX2 and Arg 180, Ile 184, His 280 of Aurora A (Figure 3C). Strong interactions formed by withanone with these particular residues would result in building steric as well as thermodynamic barriers to the association of the two subunits, thus providing hindrance for binding of TPX2 to Aurora A at these particular residues. This obstruction would pave way to unlikely complex formation capability yielding in either the formation of a deformed complex or at extreme no complex formation at all. Moreover, the deformed complex would be thermodynamically much less stable as compared to the native complex owing to the non-availability of withanone occupied hydrophobic interaction forming residues of Aurora A. In both cases, the TPX2-Aurora A complex would not get assembled to its catalytically active form. These non-covalent interactions help stabilize the binding of the ligand with the macromolecule by lowering the energy of the system. The absence of active dual subunit complex would result in disposition of Aurora A to autophosphorylation and inhibitory action of PP1. This would ultimately lead to non-translocation of Aurora A to the spindle microtubules as the poles, thus arresting its nefarious acts.

### Flexible docking of withanone into active TPX2-Aurora A complex

**Figure 4:**
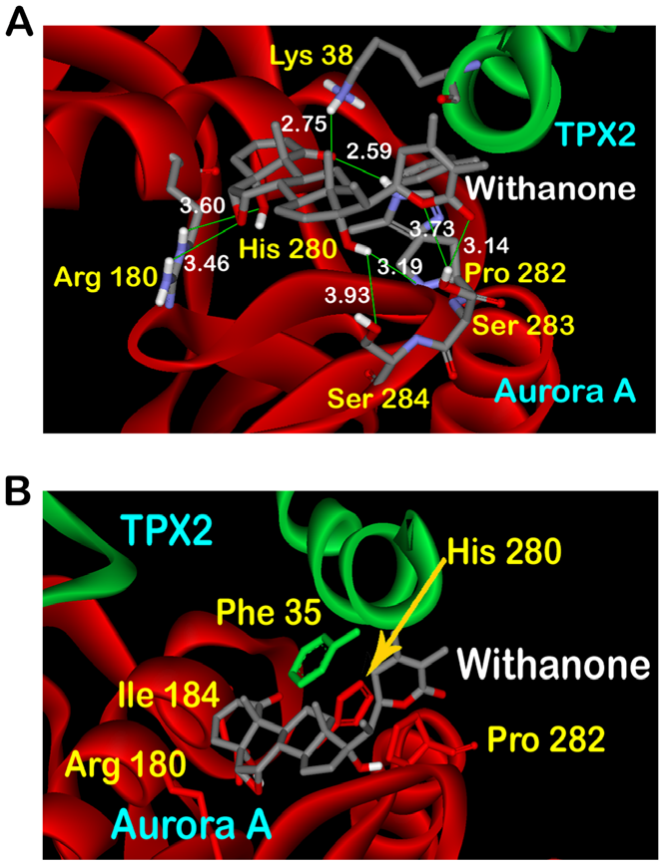
Interactions of docked withanone with flexible receptor. (A) The docked withanone forming intermolecular interactions (B) Withanone forming extensive H-bonds with the residues of flexible receptor.

Docking of withanone performed on the receptor in which the key residues responsible for holding up of the two domains were kept flexible also provided interesting results. Four clusters were obtained for Genetic Algorithm run for generation of ten models. Large negative binding energies were obtained for all the clusters as evident from Table 1. The lowest binding energy obtained using this docking mode was more than twice as low as that of the energy obtained from semi-flexible binding of withanone to the rigid receptor. In the undocked protein, the ring side chains of the two amino acids Phe 35 of TPX2 and His 280 of Aurora A were forming edge to face pi-pi interactions. But the presence of docked withanone disrupts this crucial non-covalent bonding in Cluster 1. As shown in Figure 4A, the ring group of withanone itself forms stacking intermolecular interactions with the rings of His 280 and Pro 282. This also adds to lowering of the energy of the complex as the stacking forces which are more energetically stable than the perpendicular ones. Withanone closely interacts with Arg 180 and Ile 184 also of Aurora A. The high binding energy obtained for withanone can be explained by the fact that the flexibility of the receptor resulted in formation of a very extensive H-bonding with the ligand Figure 4B. The terminal hydroxyl group and epoxy group of withanone was found H-bonding with Arg 180 with bind distances of 3.60 and 3.46 Å. The middle hydroxyl bonds with Pro 282 and Ser 284 and the ring lactone group interacts with Ser 282. The placing of withanone among these residues can be considered responsible for the enzyme inhibition, since the segment containing residues 274–299 has been described as kinase activation segment [40].

**Table 1:** Clustering results obtained from flexible docking of withanone into TPX2-Aurora A complex.

Receptor	No. of AutoDock clusters^a^^,^ b	Cluster rank^b^	No. of structures in the cluster	Lowest binding energy of cluster	Energy range within cluster
		1	3	−16.27	−16.27 to −14.95
TPX2-Aurora A complex	4 (10)	2	2	−15.65	−15.65 to −14.58
		3	4	−15.61	−15.61 to −14.35
		4	1	−14.63	−14.63 to −14.63

a Number of GA runs are shown in parentheses.

b Clustering is done with RMS tolerance of 5.0 Å.

The results obtained from docking of withanone into the active complex, with a majority of H-bond and hydrophobic interaction forming residues kept flexible, clearly showed that withanone blocks the intermolecular hydrophobic interactions between TPX2 and Aurora A at the residues which are significantly involved in formation of the active complex. The large value of binding energy for Cluster 1 (−16.27 Kcal/mol) involved in binding of withanone to the complex consolidates the thermodynamic stability of the binding. These results substantiate the hypothesis that withanone possess the potential to disarray the active complex by disrupting the stability of attachment of TPX2 to Aurora A, being accounted by hydrophobic and H-bond interactions.

### MD simulations in water

**Figure 5:**
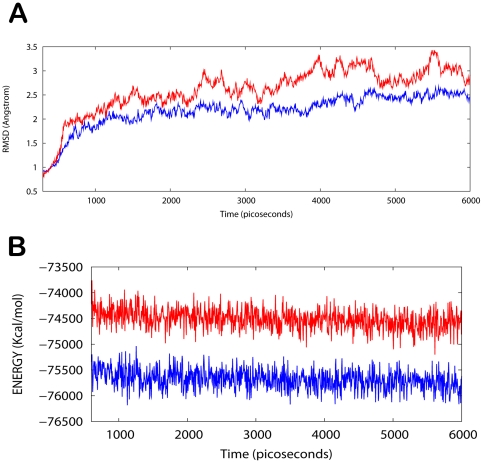
Analysis of MD trajectories. (A) Plot of root mean square deviation (RMSD) of Cα of TPX2-Aurora A (protein) and TPX2/Aurora A /withanone (complex). RMSDs were calculated using the initial structures as templates. For protein (red) the reference is the PDB structure and for complex (blue) the reference is the initial docked structure. The trajectories were captured every 2.5 ps until the simulation time reached 6000 ps. (B) Plot of total energy of TPX2-Aurora A and TPX2/Aurora A/withanone (complex). The energy trajectories of both the protein (red) and the complex (blue) are stable over the entire length of simulation time with the energy of the complex always lower than that of the protein.

The TPX2/Aurora A/withanone protein-drug binding complex with the binding energy of −7.34 kcal/mol obtained using ParDOCK was used for carrying out MD simulations. After the MD simulation, we calculated RMSDs between Cα of TPX2-Aurora A complex trajectories recorded every 1 ps and Cα of their X-ray crystal structure. The RMSDs for the trajectories of the TPX2-Aurora A complexed with withanone were also calculated using its initial PDB structure as a reference. The results in Figure 5A show that the RMSD of the complex has achieved a stationary phase during the later stage of the simulation and is always less than 2.5 Å for the entire simulation length suggesting the stability of the complex, while the RMSDs of the protein from its initial X-ray PDB structure kept increasing and is greater than that of the complex. It was also found that the energy of the complex (blue) is always lower than that of the protein alone (red) throughout the length of the simulation (Figure 5B). This rules out the possibility of the complex getting activated in the presence of ligand. The simulation lengths used in the entire study were long enough to allow rearrangement of side chains of the native as well as the drug complexed protein to find their most stable binding modes. In conclusion, the present MD simulations support the hypothesis that withanone is quite a probable and a worth small molecule ligand for targeting/inhibiting the kinase association complex. These results would be valuable for further designing non-covalent type inhibitors with high specificity and potent activity.

### Validation using experimental assays

**Figure 6:**
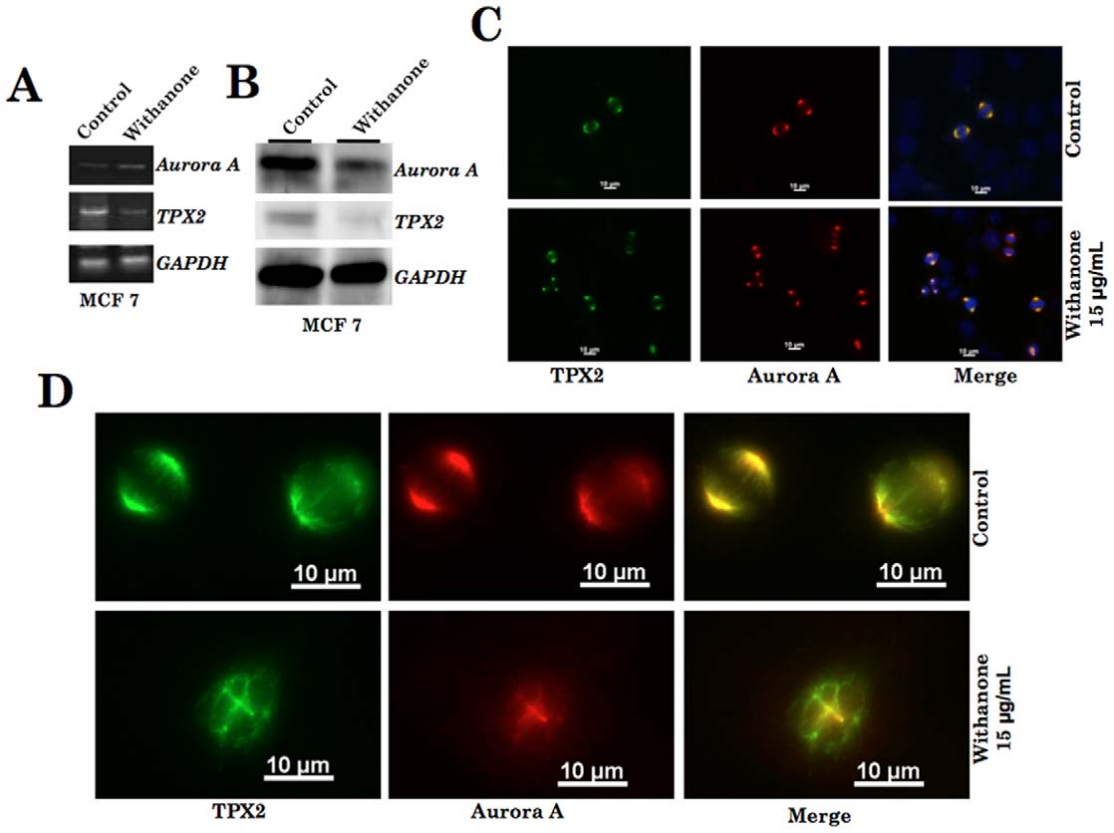
Expression analyses of Aurora A and TPX2 in control and withanone- treated human breast carcinoma (MCF 7) cells. (A) RT-PCR with gene specific primers and (B) Western blotting with specific antibodies. (C) Immunolocalization of TPX2 and Aurora A in control and withanone treated MCF 7 cells showing Aurora A (red), TPX2 (green) and co-localization of the two (yellow). (D) Representative high magnification images of TPX2 and Aurora A immunostaining showing normal and disrupted mitotic spindle in control and withanone treated cells, respectively.

**Figure 7:**
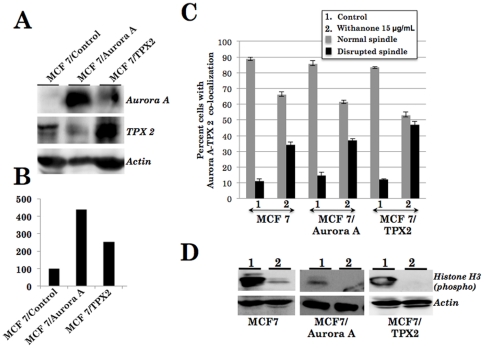
Expression analyses of Aurora A and TPX2 in control and overexpressing derivative cell lines. (A) Western blotting of Aurora A and TPX2 in control MCF 7 and its derivative overexpressing cells, quantitation of the signals is shown in (B). (C) Aurora A and TPX2 co-localization analysis in control and withanone-treated cells showing decrease in the number of cells with co-localization of the two proteins and the number of cells with disrupted mitotic spindle in withanone-treated cultures. (D) Western blotting of control and withanone-treated cells with anti-phospho histone H3 antibody showing decrease indicating the inactivation of Aurora A-TPX2 complex in withanone treated cells.

We finally validated the computational predictions using cell-based assays. Human cancer cells were first treated with withanone and the expression of TPX2 and Aurora A were examined by RT-PCR, Western blotting and immunocytostaining analyses. RT-PCR analysis of Aurora A and TPX2 in control and withanone treated MCF 7 and U2OS cells showed only a minor difference in the expression level of both mRNAs (Figure 6A and data not shown). Western blotting with specific antibodies revealed significant decrease in the protein levels of both Aurora A and TPX2 in response to withanone in the both the cell lines (Figure 6B and data not shown) examined suggesting that the decrease may reflect their enhanced degradation as a result of disruption of their complex formation as predicted by computational analysis. We next performed immunocytostaining to investigate the TPX2-Aurora A complex and mitotic spindle formation in control and withanone-treated cells. As shown in Figures 6C and 6D, withanone-treated MCF 7 cells showed decrease in the number of cells with co-localized Aurora A and TPX2 and increase in number of cells showing the disruption of mitotic spindle. Similar data were obtained in U2OS cells. We next generated derivatives of MCF 7 and U2OS cells that overexpressed either TPX2 or Aurora A (Figures 7A and 7B) and performed an extensive imaging analysis on Aurora A-TPX2 colocalization and mitotic spindle formation in both control and withanone treated cells (Figure 7C). The cells when treated with withanone showed increase in the number of cells with seggregated proteins and disrupted spindle formation (Figure 7A–C) suggesting that increase in the expression of either Aurora A or TPX2 was not sufficient to overcome the effect of withanone. Consistent to these findings, the overexpressing derivatives underwent growth arrest in response to the withanone treatment. In order to further substantiate the disruption of Aurora A-TPX2 active complex formation by withanone, we investigated the kinase activity of the complex in control and treated cells. Phosphorylation of Aurora A and its subtrate histone H3 was examined in control and withanone treated cells by using phosphorylation specific antibodies. As shown in Figure 7D, there was a significant decrease in phosphorylated histone H3 in withanone treated cells. Furthermore, Aurora A and TPX2 overexpressing cells that showed disruption of Aurora A-TPX2 complex formation (Figure 7C) and growth arrest (data not shown) in response to withanone treatment also showed similar decrease in the level of phosphorylated histone H3 (Figure 7D). These data demonstrated that withanone abrogates TPX2-Aurora A complex formation, inactivates the Aurora A-TPX2 kinase complex resulting in the disruption of the mitotic spindle formation and inhibits cancer cell division. It, therefore, could be a potential anticancer drug.

## Materials and Methods

### Ligand and receptors

The crystal structure of the TPX2-Aurora A association domain [PDB: 3E5A] [40] was obtained from the Protein Data Bank (PDB) [41]. This structure was then subjected to certain modifications which would make it suitable for docking. These included the use of repairCommands module of AutoDock to supplement a number of missing atoms, removal of water molecules to clean the crystal structure and addition of H-atoms to these target proteins for correct ionization and tautomeric states of amino acid residues. The modified structure was used for all flexible and semi-flexible docking studies. The ligand molecule withanone [PubChem: 21679027] was retrieved from NCBI-PubChem Compound database [42]. The structure of this compound is shown in Figure S1. The energy of the ligand molecule and receptors were minimized in Steepest Descent and Conjugate Gradient methods using Accelrys Discovery Studio (Version 1.7, Accelrys Software Inc.). Each of the minimization methods were carried out with CHARMm force field.

### Structural aspects of TPX2-Aurora A association complex

The structural features of the receptor macromolecule considered in our study [PDB: 3E5A] have been described in detail elsewhere [40] by the depositors of the crystal structure to the Protein Data Bank. Briefly, the structure includes the catalytic core of the Aurora A kinase (residues 123–387) and two segments of TPX2 (residues 6–21 and 26–43). The four missing residues of TPX2 (residues 22–25) have not been modelled due to the absence of ordered electron density. This fragment of TPX2 (residues 1–43) is independently capable of binding, kinase activation, and protection from dephosphorylation of Aurora A kinase. Furthermore, Aurora A shows increased catalytic efficiency due to the addition of TPX2 (residues 1–43) [43]. The Aurora A catalytic core (residues 123–387) comprises of an N-terminal lobe (residues 123–210) and a large C-terminal lobe (residues 217–387). The active site is located at the interface between the lobes which includes the ATP binding site, the catalytic base (Asp256), and the kinase activation segment (residues 274–299). The binding of TPX2 with Aurora A is in two stretches, similar to that in TPX2/Aurora A/ADP structure [11]. The binding of upstream stretch (residues 6–21) to the N-terminal lobe of Aurora A is in a mostly extended conformation while the downstream stretch (residues 26–43) forms an α-helical conformation that interacts with both helix αC and the activation segment of Aurora A. The two Aurora A binding motifs of TPX2 are connected by a flexible linker (disordered in the structure). The conserved residues Tyr8, Tyr10, Ala12, Phe16, Ile17, Phe19, Trp34, Phe35 and Ala39 of TPX2 interact closely with the Aurora A catalytic core.

### Semi-flexible docking

AutoDock 4.0 suite was used as molecular-docking tool in order to carry out the docking simulations [44]. Several studies report the comparison of AutoDock with other docking programs. AutoDock has been found to be able to locate docking modes that are consistent with X-ray crystal structures [45], [46]. AutoDock helps to simulate interactions between substrates or drug candidates which act as ligands and their macromolecular receptors of the known three dimensional structures. AutoDock allows full ligand flexibility, more than that described anywhere else [44]. In our docking simulations we used the TPX2-Aurora A association domain structure to perform semi-flexible docking, with the ligand withanone made flexible while keeping the receptor macromolecule rigid. The flexibility of ligand molecule gives it the freedom to search from six degrees of rotational and translational freedom and an arbitrary number of torsional degrees of freedom. A random perturbation to each is applied at each time step, and the interaction energy was evaluated for the new location and conformation [47].

### Flexible docking

One of the characteristic features of AutoDock 4.0 is that it allows side chains in the proteins to be flexible and can optionally model flexibility in target macromolecule. Thus, flexibility of receptor molecule was also exploited in docking studies by making use of AutoDock flexres scripts. Our simulations are based on assumption that the ligand which arrives at the binding site formed by the two chains of receptor molecule would try to associate with them in order to minimise the energy of the system. The key residues of Aurora A and TPX2 chains which form critical interactions with the corresponding residues in the two chains as reported [40] were made flexible to prove the above said assumption. This helped us in observing different interaction modes of receptor with the ligand as well as the rearrangements that follow. The peptide bonds of the amino acids whose side chains were kept flexible were kept ‘inactive’ and ‘non-rotatable’.

The graphical user interface program “AutoDock Tools” was used for preparing, running and analyzing the docking simulations. Protein structure used for docking simulations was prepared by attributing Kollman united atom charges, solvation parameters and polar hydrogens to the receptor PDB structure. Water molecules were removed from this structure to make it a free receptor. Ligands which are not peptides were first assigned a Gasteiger charge and then non-polar hydrogens were merged. The use of AutoDock for simulations saves time as it assigns rigid roots to the ligand automatically as compared to manual picking. Four bonds in the ligand were made “active” or rotatable. Default solvation parameters were assigned to the receptor. The energy scoring grid was prepared as a 40, 40 and 32 A° (x, y, and z) cube. The spacing between grid points was 0.375 A°.

### Selection and representation of docking modes

The Lamarckian Genetic Algorithm (LGA) was chosen to search for the best conformers. The best docking solution (minimum docked free energy) is reported by AutoDock for each GA run. Total number of clusters and the rank of each docking mode (cluster rank) are also reported in the cluster analysis performed by AutoDock. Docking modes were selected on the basis of two criteria: extent of ligands' associations with the key residues of the receptor and the thermodynamic stability of the docked complex so obtained. The lowest energy docking mode that would conform to the above said two parameters was selected from over 10 GA run and hence 10 total docking modes. All the AutoDock docking runs were performed in Intel Core 2 Duo P8400 CPU @ 2.26 GHz of Sony origin, with 3 GB DDR RAM. AutoDock 4.0 was compiled and run under Windows VISTA operating system. The output from AutoDock and all modeling studies as well as images were generated with PyMOL [48] and Accelerys ViewerLite 5.0. The hydrogen bonds lengths were measured between the hydrogen and its assumed binding partner using ViewerLite.

### Confirmation of the docking results

The docking results obtained using AutoDock were also confirmed using ParDOCK [49], which is an all atom energy based monte carlo docking protocol. Docking using ParDOCK requires a reference complex (target protein bound to a reference ligand) and a candidate molecule along with specific mention of the centre of mass of the cavity on which the ligand is to be docked.

### MD simulations in water

The AMBER v.11 package [50] was used to prepare the protein and the ligand files as well as for the Molecular Dynamics (MD) simulations. The binding complexes of TPX2/Aurora A/withanone obtained using ParDOCK and the un-docked TPX2-Aurora A association protein simulated in this study were neutralized by adding appropriate number of sodium counter-ions and were solvated in a octahedron box of TIP4PEW water with a 10 Å distance between the protein surface and the box boundary [51]. The partial atomic charges for the ligands were obtained after optimization at the Hartree-Fock level with 6–31G* basis set and subsequent single-point calculation of the electrostatic potential to which the charge were fitted using RESP procedure [52], [53]. Force field parameters of the ligands were assigned based on the atom types of the force field model developed by Cornell et al [54]. The binding complex was effected with a 750 step minimization using SANDER module of AMBER in the steepest descent followed by a 250 step minimization in conjugate gradient. Then the system was equilibrated beginning with the protein atom restrained simulations having 200 ps equilibration dynamics of the solvent molecules at 300 K. Next step involved the equilibration of the solute molecules with a fixed configuration of the solvent molecules in which the system was slowly heated from T = 0 to 300 K in 60 intervals each involving heating for a 5 K increase in 2.5 ps followed by an equal time duration equilibration step. The entire system was then equilibrated at 300 K for 200 ps before a sufficiently long MD simulation for 6 ns at room temperature. The MD simulations were performed with a periodic boundary condition in the NPT ensemble at T = 298.15 K with Berendsen temperature coupling [55] and constant pressure P = 1 atm with isotropic molecule-based scaling. We used a time step of 2 fs and a non-bond interaction cutoff radius of 10 Å. MD simulations were performed on a 320 processors SUN Microsystems clusters at Supercomputing Facility (SCFBio) at IIT Delhi.

### Cell culture and treatment with withanone

Human osteosarcoma cells (U2OS) [56] obtained from American Type Culture Collection (ATCC) and breast carcinoma cells (MCF7) [56] obtained from Japanese Collection of Research Bioresources (JCRB) were cultured in Dulbecco's modified Eagle's minimal essential medium (DMEM), supplemented with 10% fetal bovine serum at 37°C, in an atmosphere of 5% CO2 and 95% air in a humidified incubator. Cells were treated with withanone at about 60–70% confluency and harvested for following analyses after 48 h of the treatment. Aurora A and TPX2 overexpressing MCF 7 and U2OS cells were generated by using retrovirus (pBABE-puro/Aurora A) and plasmid (pPAGFP-C1/TPX2 ) expression vectors obtained from Addgene (#8510:hAur-A, gift of Joan Ruderman and #31226: TPX 2, gift of Patricia Wadsworth).

### Aurora A and TPX2 expression analysis

Total RNA from the control and withanone treated cells was isolated with the RNeasy Mini Kit (QIAGEN). First-strand cDNA was synthesized using 1 µg RNA with oligo dT as the primer with MMLV reverse transcriptase. PCR (denaturation-95°C:1 min, annealing-54°C:1 min and extension-72°C:1 min for 30 cycles) was performed using specific primers for (i) Aurora-A ( sense 5′-agctccagttggaggtccaaaac-3′ and antisense 5′-gcctggagacaggatgaggtaca-3′) and (ii) TPX2 (sense 5′-AATGCATCTTCCCCAGAGAAAGC-3′ and antisense 5′-TTCATTCTTTTTCCGCATCTCCA-3′. 7 µl of each PCR product (total 30 µl) was used for electrophoresis on a 1% agarose gel, stained with ethidium bromide, visualized with UV light and photographed (Bio-Rad). For proteins analysis, control and withanone (10–15 µg/ml) treated cells were lysed in were lysed in NP-40 lysis buffer [10 mM Tris-HCl (pH 7.4), 150 mM NaCl, 5 mM EDTA, 1% NP-40] supplemented with a protease inhibitor cocktail (Complete Mini; Roche Diagnostics K.K., Basel, Switzerland). Aliquots of 20 µg of total protein were resolved on a SDS-polyacrylamide gel electrophoresis (SDS-PAGE), transferred to a polyvinylidene difluoride membrane (Atto Corporation, Tokyo, Japan) and were probed with anti-Auroa A (polyclonal, TransGenic Inc) and TPX2 (monoclonal clone18D5-1, Abcam) antibody. Anti-actin antibody (Chemicon International, USA) probing was used as loading control. Horseradish peroxidase-linked antibodies against rabbit and mouse IgG raised in donkey (GE Healthcare Bio-Science AB, Uppsala, Sweden) were used as secondary antibodies. Immunoreactive bands were detected with enhanced chemiluminescence reagent (GE Healthcare Bio-Science AB) and LAS-3000mini luminoimage analyzer (Fujifilm Corporation, Tokyo, Japan) following the manufacturer's instructions. Quantification of immunoreactive bands was performed with Image Gauge software (Fujifilm Corporation, Tokyo, Japan).

### Immunocytostaining

For immunostaining, cells were plated on glass coverslips placed in a 12-well culture (10^4^ cells /coverslip). After 24 h, when cells had attached to the surface and spread well, they were treated with withanone for 48 h following which cells were washed with cold PBS three times and then fixed with pre-chilled methanol/acetone (1∶1) mixture for 5 min. Fixed cells were washed with PBS twice, permeabilized with 0.5% Triton X-100 in PBS for 10 min and blocked with 0.2% BSA/PBS for 10 min. They were incubated with the anti-Aurora A and TPX2 antibodies for 1 h at room temperature, washed three times with 0.2% Triton X-100 in PBS, and then incubated with secondary antibodies (Alexa-594-conjugated goat anti-rabbit and Alexa 488-conjugated goat anti-mouse, Molecular Probes). After extensive washings with 0.2% Triton X-100 in PBS, cells were examined on a Carl Zeiss microscope (Axiovert 200 M).

## Supporting Information

Figure S1 **Structures of withanolides.** (A) Withanone falls under the family of compounds known as withanolides which are a group of naturally occurring C28- steroidal lactones built on an intact or rearranged ergostane framework, in which C-22 and C- 26 are appropriately oxidized to form a six-membered lactone ring. The basic structure is designated as the withanolide skeleton defined as a 22-hydroxyergostan-26-oic acid-26,22-lactone. (B) Structure of withanone.
(JPG)
