# Supplementary figures and images for: Ashwagandha Derived Withanone Targets TPX2-Aurora A Complex: Computational and Experimental Evidence to its Anticancer Activity
Source: PLoS One. 2012 Jan 27;7(1):e30890. doi: 10.1371/journal.pone.0030890 (PMC3267739; doi:10.1371/journal.pone.0030890)

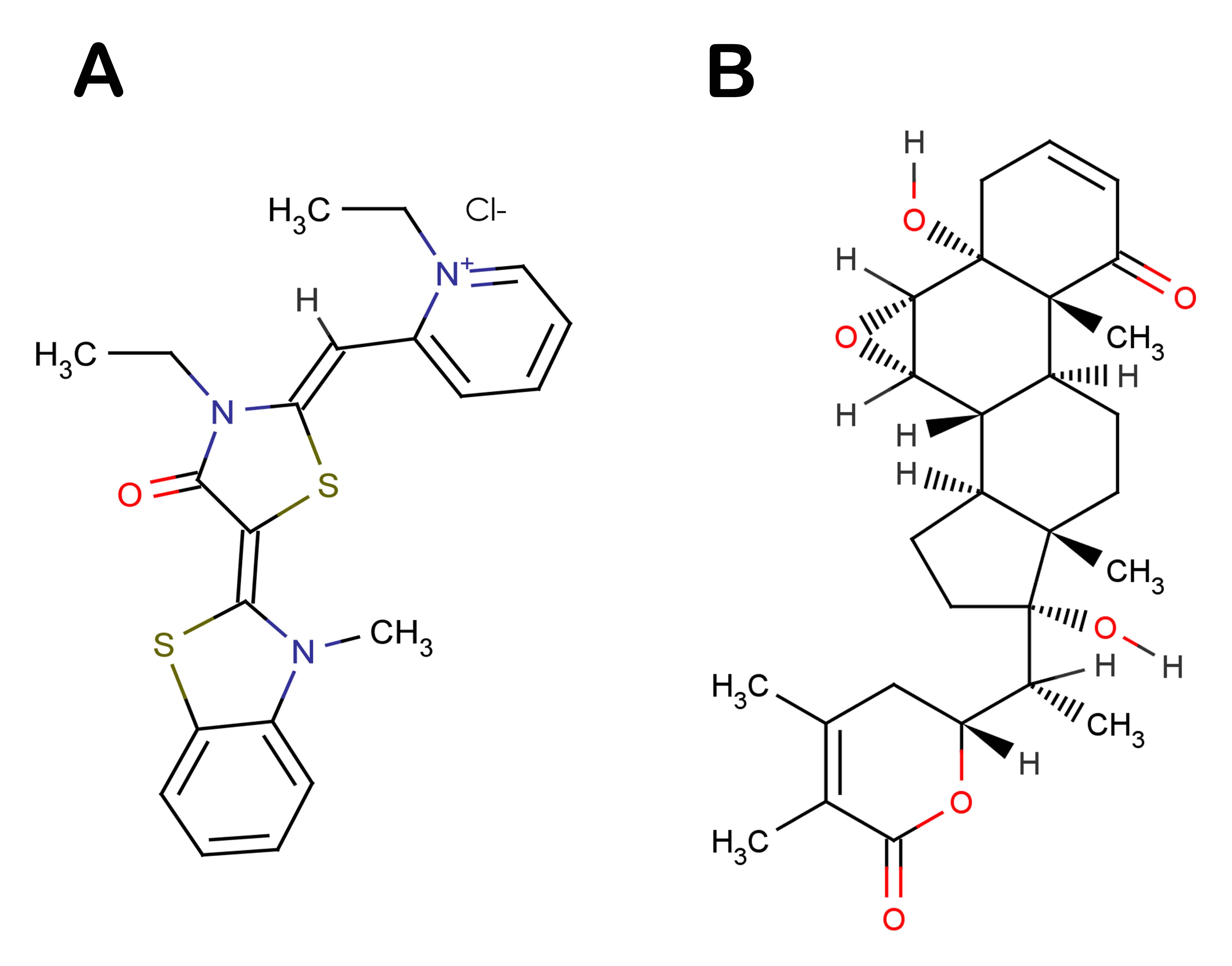

Supplement: Figure S1 — Structures of withanolides. (A) Withanone falls under the family of compounds known as withanolides which are a group of naturally occurring C28- steroidal lactones built on an intact or rearranged ergostane framework, in which C-22 and C- 26 are appropriately oxidized to form a six-membered lactone ring. The basic structure is designated as the withanolide skeleton defined as a 22-hydroxyergostan-26-oic acid-26,22-lactone. (B) Structure of withanone. (JPG) [file pone.0030890.s001.jpg]
